# The Effect of Visual Cues on Auditory Stream Segregation in Musicians and Non-Musicians

**DOI:** 10.1371/journal.pone.0011297

**Published:** 2010-06-23

**Authors:** Jeremy Marozeau, Hamish Innes-Brown, David B. Grayden, Anthony N. Burkitt, Peter J. Blamey

**Affiliations:** 1 The Bionic Ear Institute, Melbourne, Australia; 2 University of Melbourne, Melbourne, Australia; Center for Genomic Regulation, Spain

## Abstract

**Background:**

The ability to separate two interleaved melodies is an important factor in music appreciation. This ability is greatly reduced in people with hearing impairment, contributing to difficulties in music appreciation. The aim of this study was to assess whether visual cues, musical training or musical context could have an effect on this ability, and potentially improve music appreciation for the hearing impaired.

**Methods:**

Musicians (N = 18) and non-musicians (N = 19) were asked to rate the difficulty of segregating a four-note repeating melody from interleaved random distracter notes. Visual cues were provided on half the blocks, and two musical contexts were tested, with the overlap between melody and distracter notes either gradually increasing or decreasing.

**Conclusions:**

Visual cues, musical training, and musical context all affected the difficulty of extracting the melody from a background of interleaved random distracter notes. Visual cues were effective in reducing the difficulty of segregating the melody from distracter notes, even in individuals with no musical training. These results are consistent with theories that indicate an important role for central (top-down) processes in auditory streaming mechanisms, and suggest that visual cues may help the hearing-impaired enjoy music.

## Introduction

Music often contains multiple “streams”–a number of melodic lines for instance–either played on the same or separate instruments. In order to enjoy music, listeners must be able to perceptually separate and group auditory streams. This ability is called auditory stream segregation, and is based partly on perceptual differences (such as pitch and timbre) between the streams [1], [2], [3]. Unfortunately, these perceptual cues are degraded by hearing loss [4] and hearing devices such the cochlear implant [5], [6], [7], [8], leading to poor auditory stream segregation [9], [10], [11], [12], [13], and adding to the already problematic issue of music appreciation for CI users.

However, recent research [14], [15] has emphasised the fact that as well as relying on these “bottom-up” signals from the peripheral auditory system, successful stream segregation also involves more “top-down” or feedback processes. These top-down effects can be guided by processes such as memory, expectation or attention. Visual cues [16] and training in music [17] have also both been found to improve the ability to segregate simple sounds from auditory backgrounds. Therefore, such top-down effects might help hearing-impaired listeners to restore their ability to perceptually isolate a melodic line from a complex musical sequence. In order to test this hypothesis, it is first necessary to evaluate how melody separation interacts with top-down effects such as the addition of visual cues, music training, and musical context in listeners with normal hearing. This paper reports two experiments that studied such interactions.

### Effects of Vision

The power of visual cues to improve auditory perception has long been known, particularly in the case of speech perception in background noise. When a speaker's lip and facial movements are visible, an improvement in performance equivalent to increasing the signal-to-noise ratio by up to 15 dB has been observed [18]. Visual stimuli can also affect perception in other auditory tasks. For instance, presentation of a visual stimulus can increase the perceived loudness of white noise [19], and discriminations of pitch and loudness improve when presentation of a concurrent visual stimulus matches the features of the sound [20]. When high-brightness visual stimuli were paired with high-pitch or high-loudness sounds, auditory discriminations were improved compared to when the pairing was incongruent.

Visual stimuli can also influence how sounds are grouped perceptually. In an early study investigating the interplay between auditory and visual grouping, O'Leary and Rhodes [21] studied the effect of visual streaming on auditory streaming, and vice versa. The auditory stimuli were sets of low(A)-high(B)-low(A) tones–a typical auditory streaming paradigm–that can either be perceived as integrated in a single stream (A-B-A--A-B-A: a “galloping” percept), or segregated into two streams (A-A--A-A and B----B: a “Morse code” percept). The visual analogue of this paradigm also exhibits “streaming” effects. When groups of dots placed at high and low positions on a screen are presented alternately, they can either be perceived as integrated (in which case they appear to move up and down), or segregated (in which case the high and low dots appear to flash). In both cases, the likelihood of perceiving the stimuli as segregated or integrated can be influenced by altering the rate of presentation as well as the separation in frequency (for auditory stimuli) or height (for visual stimuli) between the two streams. In their experiment, O'Leary & Rhodes [21] determined the ranges of rate and separation parameters in which both the visual and auditory streams were perceived as integrated and segregated. The auditory stimuli were then presented using parameters for which the perception was ambiguous (either streaming or integrated), and the visual stimuli were presented using parameters chosen to strongly induce a segregated percept. The experiment was repeated with ambiguous visual stimuli and segregated auditory stimuli. The authors found that in both cases, clearly segregated stimuli in one modality could increase segregation of ambiguous stimuli in the other modality, with segregated visual stimuli affecting auditory streaming more strongly than vice versa. Pressnitzer & Hupe [22] have also demonstrated that visual and auditory bistable perceptions have many properties in common. The hallmark criteria for visual bistability (exclusivity, randomness, and inevitability) were all shown to be met for both an auditory and visual stimulus sequence. Although this suggests that common principles might underlie bistable perception in both modalities, there was no correlation between bistable perception in each modality, suggesting that these common principles are independently implemented in each sensory modality.

More recently, Rahne *et al* [16] used the mismatch negativity potential (MMN–a scalp electrical response recorded in response to violations of an expected sound sequence) to show a similar effect. In their experiment, participants listened to a set of tones making up two possible streams. Similar to O'Leary & Rhodes [21], the frequency separation of the two streams, as well as the rate at which they were presented, was chosen such that the stimuli were ambiguous - participants reported either one (integrated) or two (segregated) streams. Two possible visual cues were then paired to the sounds. A series of shapes presented with each note matched either the integrated or segregated perception. To test the effect of the visual cue, an occasional “deviant” sequence was introduced in one of the two auditory streams and the MMN recorded in response to the deviant tones. In order to perceive the deviant tone, and elicit the MMN, it was necessary for the auditory stimuli to be perceived as two streams. They found that MMN was present only when the visual stimuli coincided with the *segregated* perception. Thus, the visual cue was found to improve the ability to segregate two streams of ambiguously organised tones. However, it is unknown whether this benefit extends to segregation in music, such as in the segregation of a melody from background notes.

### Effect of Music Training

Musical education is an intense training activity–professional musicians spend many hours per day listening to and producing multiple streams of auditory information. This intense activity, usually over the course of many years, has a variety of effects on behaviour, brain structure and brain function. A combined magnetoencephalography and structural MRI study [23] has shown that musical aptitude is correlated with both the gray matter volume of Heschl's gyrus (a structure containing the primary auditory cortex) as well as tone-evoked neural activity in this gyrus. Musicians also show faster responses and enhanced representation of pitch and timbre in the brainstem to music and speech stimuli [24]. Importantly, these improvements were larger still when musically-trained participants simultaneously lip-read or watched videos of a musician playing, suggesting that visual information may improve representations of pitch at brainstem level as well as cortical level, and that musicians are more able to utilise visual cues to enhance their perception of auditory stimuli.

Training in music has also been shown to influence auditory stream segregation. The decay of streaming effects occurs more slowly in musicians compared to non-musicians [25], and in conditions with reduced spectral complexity, musicians can separate streams of tones that are closer in pitch than non-musicians [26]. Listeners with musical training are also better able to separate concurrently presented sounds. Zendel & Alain [17] presented musically trained and untrained listeners with a series of complex tones. When the second harmonic in these tones was deliberately mistuned, musicians perceived the tones as segregated into two streams more often than non-trained listeners. The authors also found evidence from EEG recordings made during the task that the musicians' improvement in detecting the mistuned harmonics was due to changes in early perceptual processing in addition to higher level cognitive processes.

### Effect of Music Context

Musical phrases are heard within a specific context. For example, a specific melodic line may be heard against a variety of different backgrounds–sometimes appearing out of the background and sometimes disappearing into it. Depending on the prior knowledge of the listener, and on the specific arrangement of the musical background, a melody can be perceived in different ways. Listeners with more experience may be able to maintain their perception of a melody as it becomes more entangled in accompanying notes and may be able to identify it earlier as it emerges. The emergence of the melody can be influenced by the separation in pitch of the melody and background notes. Dowling [27] showed that pairs of interleaved familiar melodies could be identified, but only when the pitch range of the distracter notes did not overlap with the melody. If the listeners were told in advance which melody to search for, however, the interference of the background notes could be partly overcome. In the initial experiment, the pitch overlap of the two melodies was gradually *decreased* until participants were able to name the melodies (generally at the point where there was no longer any overlap). However, when two high-performing participants repeated the experiment with the overlap gradually *increasing*, they were able to continue following a single melody even when the melodies completely overlapped. Unfortunately, apart from noting this effect in these two participants, Dowling did not test the effect on stream segregation of increasing or decreasing the overlap between the melodies. It would seem reasonable to assume that listeners should be able to better segregate the melody from the background notes in the *increasing* context compared to the *decreasing* context due to the longer exposure to the segregated melody. However, recent studies by Snyder *et al*. [28], [29]report a contrastive context effect in an ABA auditory streaming paradigm. In these studies, as expected, the listeners likelihood to perceive the sequence ABA as two streams increased with the frequency difference (Δƒ) between A and B. However, further analysis of the results showed a significant effect of the Δƒ presented in the previous trial: if the Δƒ of the previous trial was larger than the Δƒ of the current trial, the listener's likelihood to segregate the two tones decreased. In Dowling's increasing context, the overlap note difference between the two melodies gradually increased, therefore the context effect described by Snyder *et al*. should accelerate the fusion likelihood between the melodies.

### Overview of the Study

**Figure 1:**
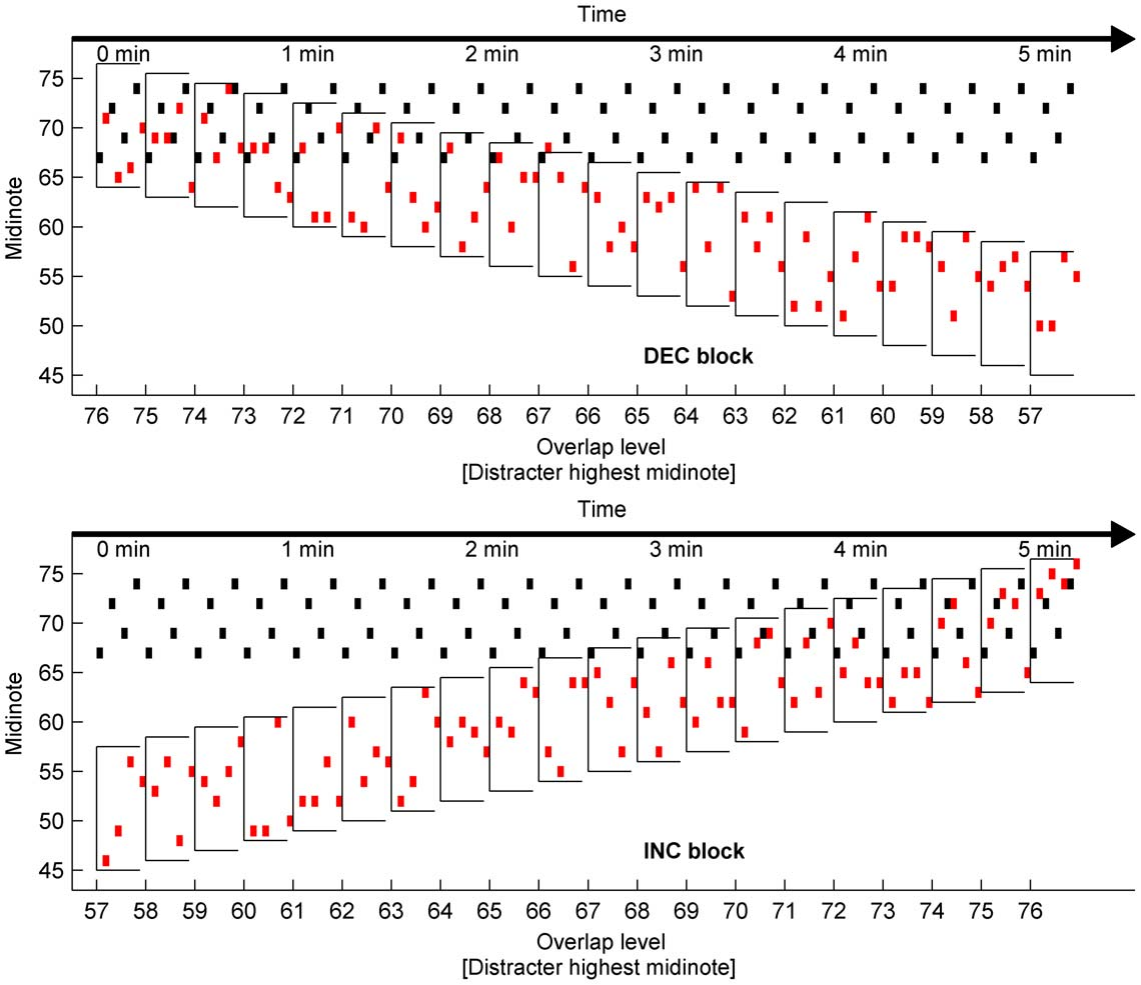
Procedure of Experiment 1. A decreasing (upper panel) and an increasing (lower panel) block are shown. The melody notes (black/dark dots) play continuously. Distracter notes (red/light dots) are interleaved with the melody notes, and are selected from a range of 12 consecutive midinotes (an octave). The separation is increased or decreased by one midinote per level, for 20 levels. Within each level, the melody is repeated 20 times (a single melody is shown).

In the present experiment, the effect of visual cues, musical training and musical context on musical streaming were examined in normal-hearing listeners. A musical streaming paradigm was employed that involved the extraction of a simple repeating melody from a background of interleaved random distracter notes (see Figure 1). The melody spanned 8 semitones, while the distracter notes were pseudo-randomly chosen from an octave-wide pool of 12 semitones. The overlap between the melody and distracter notes was altered by changing the range of possible distracter notes. In increasing blocks (INC), the overlap between melody and distracter notes gradually increased in 20 steps of 1 semitone (starting from an octave separation and increasing towards complete overlap). As the overlap increased, it became gradually more difficult to segregate the melody from the distracter notes. In decreasing blocks (DEC), the overlap was gradually decreased in 20 steps of one semitone (starting from completely overlapped, and decreasing to one octave separation). As the overlap decreased, the melody became gradually less difficult to segregate. Comparing increasing (INC) and decreasing (DEC) blocks allowed a comparison of two musical contexts: in the INC blocks, the melody is first salient (easily segregated) and then gradually became masked (melody and distracter streams were fused); in DEC blocks the melody is initially masked and then gradually emerges from the background. As the melody and distracter notes were playing, participants were asked to continuously rate the difficulty of extracting the melody, using a variable slider with a scale marked from “no difficulty” to “impossible” (see methods section). The difficulty of extracting the melody was measured with and without presentation of a concurrent visual cue, which consisted of a visual representation of the pitch of each melody note and the time at which it played. Musicians and non-musicians were tested. We hypothesised that

1] Visual cues would reduce the difficulty of extracting a simple melody from random distracter notes
2] Musicians would find the task less difficult overall and benefit more than non-musicians from the visual cues
3] The musical context would affect the result as it should be easier to segregate a melody in the INC condition than the DEC condition.

If visual cues decrease the difficulty of segregating a melody from background notes, it may be possible to design an appropriate visual cue for use by the hearing impaired in order to improve their ability to appreciate music. It is unknown whether musical training will be required to utilise the visual cue. If musicians gain more benefit from the visual cue, training using the device may transfer to everyday listening situations.

## Results

### Experiment 1

#### Classification of participants into Musician or Non-Musician groups

The criteria for defining ‘musicians’ vs ‘non-musicians’ is variously defined on the basis of years of experience [30] or main occupation [31], or it can be indeterminate [32]. A complicating factor in defining groups based on a single measure is the great variety of activities that can contribute to musical training–using a single measure may not capture the extent of musical training accurately for all individuals. Excluding individuals with any amount of ‘musical training’ from the non-musician group is also problematic due to the relative scarcity of individuals who have had absolutely no experience of musical training. Most people have had at least some musical training (for instance in primary school). In order to classify the 37 participants as musicians or non-musicians more objectively, the participants were divided into two groups according to a hierarchical cluster analysis designed to maximise the group differences on four normalised musical activity variables: 1] sight-reading ability self-ratings, 2] general musical aptitude self-ratings, 3] the number of hours of musical practice per week, and 4] years of musical training. The cluster analysis was constrained to two possible solutions. The group composed of participants with higher scores on the musical evaluation form was designated “Musicians” (N = 18), with the remainder “Non-musicians” (N = 19). The means and standard deviations of the musical activity variables separated by the results of the cluster analysis are summarised in Table 1.

**Table 1:** Participant details.

Mean Scores (SD)	Non-musicians N = 19 (8 females)	Musicians N = 18 (9 females)
Sightreading self rating	1.6(1.9)	4.4(1.1)
Aptitude self rating	1.0(1.3)	4.3(.8)
Hours practice	1.5(3.4)	17.1(10.8)
Years playing	4.9(5.4)	24.2(6.3)

Music training details for musicians and non-musicians.

#### Raw Data

**Figure 2:**
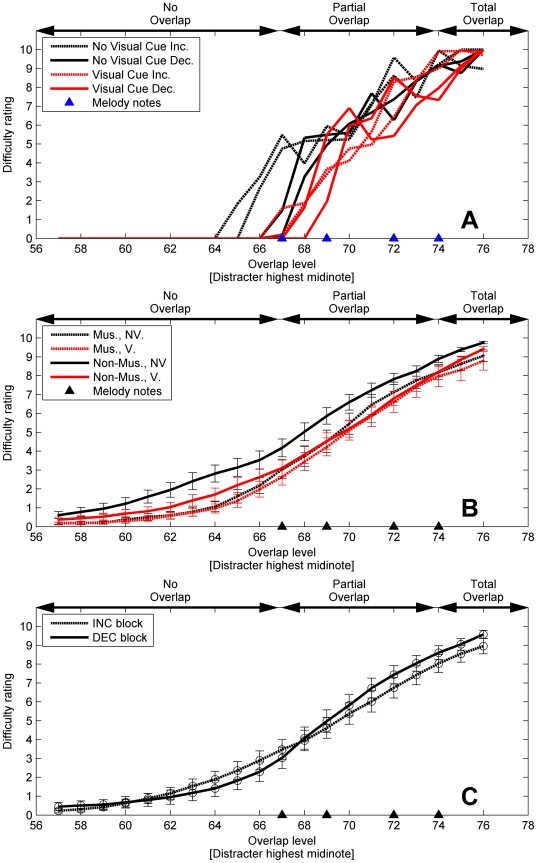
Results of the difficulty ratings (Experiment 1) as a function of the overlap level. The four notes of the target melody are represented by filled triangles. A] Difficulty ratings for one participant of the four non-visual sessions (black lines) and the four visual sessions (red lines). B] Difficulty ratings for musicians (dotted lines) and non-musician (solid lines) for sessions with visual cues (V) and without visual cues (NV) averaged across repetition and context: error bars show one standard error. C] Difficulty ratings for INC (dotted) and DEC (solid) blocks averaged across repetition and groups.

Figure 2A shows the individual results of one participant (non-musician) for illustrative purposes. The melody segregation task was run twice in each condition: with visual cues and increasing overlap (dotted red lines), no visual cues and increasing overlap (dotted black lines), visual cues and decreasing overlap (solid red lines), and no visual cues and decreasing overlap (solid black lines). When there was no overlap, the difficulty was rated as low; *i.e.*, the melody was perceived as segregated. The difficulty ratings were generally higher with higher levels of overlap. Finally, when the distracter range totally overlapped the melody (the highest distracter note was above midinote 74), the melody was judged as impossible to perceive. All participants showed a similar pattern.

#### Average Data

Figure 2B shows the average difficulty ratings for musicians and non-musicians as a function of the overlap level (expressed as the highest midinote value of the distracter note range). The ratings are averaged across INC and DEC blocks, and shown with and without the visual cue. When no visual cue was present (black lines), musicians (dashed lines) generally rated the melody segregation as less difficult than non-musicians (solid lines) across a wide range of overlap levels. However, when the visual cue was present (red lines), difficulty ratings for musicians and non-musicians were very similar. Interestingly, Figure 2B shows that the visual cues helped the non-musicians to reach about the same difficulty rating level as the musicians (14% lower difficulty ratings averaged across all overlap levels). Figure 2C shows the average rating for the INC and DEC conditions. In DEC blocks, difficulty ratings were generally higher than in INC blocks while the melody and distracter notes overlapped. The DEC blocks show overall a steeper slope of rating difficulty as function of overlap.

#### ANOVA

In order to asses the significance of the effects of visual cues, musical training and musical context on the difficulty of extracting the simple melody from the distracter notes, the difficulty ratings were entered into a repeated-measures mixed ANOVA with a between-groups factor Group (Musicians, Non-Musicians), and within-groups factors for Vision (Vision, No-vision), Context (INC, DEC), Repeat (first, second), and Overlap (20 overlap levels, from complete overlap to one octave separation). See methods section for detailed descriptions of each factor. Mauchley's test was used to estimate sphericity. Greenhouse-Geisser corrected *p* levels and estimates of sphericity (ε) are reported if Mauchley's test was violated. Table 2 summarizes the results of the analysis.

**Table 2:** ANOVA results.

	Group	Visual Cues	Context	Repeat	Level
**Group**	n.s.				
**Visual Cues**	*F* [1], [35] = 7.7, p = .009	*F* [1], [35] = 21.6 *p*<.0001			
**Context**	n.s.	n.s.	n.s.		
**Repeat**	n.s.	n.s.	n.s.	n.s.	
**Level**	n.s.	n.s.	*F*[19,665] = 8.9, *p*<.0001, *ε* = .19	n.s.	*F*[19,665] = 485, *p*<.0001, *ε* = .13

Significant main effects (diagonal) and first order interactions (lower triangle) for difficulty ratings. Greenhouse-Geisser corrected *p* levels and estimates of sphericity (*ε*) are reported if Mauchley's test for sphericity was violated.

As expected, a significant main effect of Overlap was found indicating that as the overlap between the melody and the distracter increased, difficulty ratings increased significantly. A significant main effect of the factor Vision was found, indicating that, overall, difficulty ratings were significantly lower when the visual cue was present. A significant Vision-by-Group interaction was found, indicating that difficulty ratings were significantly reduced to a greater extent for non-musicians than musicians when the visual cue was present. Furthermore, pairwise comparisons found no significant reduction in difficulty for non-musicians when the visual display was present (p = .11), and a highly significant reduction for non-musicians (p<.001).

There was also a significant Context-by-Overlap interaction, indicating that difficulty ratings varied across overlap levels differently depending on the context (INC or DEC). This indicates that when the distracter notes overlapped the melody, difficulty ratings were different for a given overlap level, depending on the context. No significant main effect or first-order interaction was found for the factor Repeat, indicating that difficulty ratings were consistent within INC and DEC blocks, no matter whether they were presented first or second.

### Experiment 2

**Figure 3:**
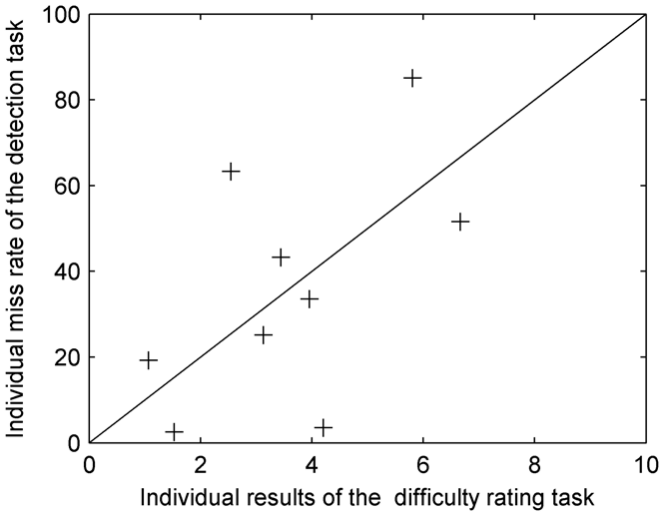
Scatter plot of average difficulty ratings in Experiment 1 vs. average miss rate in Experiment 2 for nine individuals. The line shows the identity function.

In Experiment 1, listeners were asked to rate their subjective perception of the difficulty of the task. The rating results thus may have included a component related to the response bias of the listeners. It is possible that a listener may over- or under-estimate the true difficulty. In order to validate the method, a control experiment was performed with 9 of the participants. For the sake of brevity, only a summary of the method and results is reported here. The stimuli and procedure were similar to the Experiment 1, except that: 1] an inversion of two notes of the melody was pseudo-randomly inserted, 2] the task was to report detections of the inversion with a button-press, and 3] only Non-Visual conditions were tested. It was assumed that if the listeners were able to segregate the melody, the detection task would be easy when there was no overlap between melody and distracter, and would gradually become difficult as the overlap was increased. Average difficulty ratings from Experiment 1 were compared with the miss rate (number of misses divided by the number of inversions) for melody inversions. Figure 3 plots the average difficulty rating (across all overlap levels) in Experiment 1 against the miss rate in Experiment 2. The black line shows the identity function, along which the results could be expected if participants performed similarly in the two experiments. A point below the identity line indicates a conservative response bias (i.e. the melody was judged as difficult to hear in Experiment 1, but inversions were detected well). A point above the line indicates a more liberal response bias (i.e. the melody in Experiment 1 was judged as easily perceivable, but melody inversions were not detected). Results show no overall bias, with six listeners out of nine showing very consistent responses across experiments. Since stream segregation is a necessary condition to detect melody inversions, Experiment 2 shows that difficulty ratings in the original task were a reliable indicator of stream segregation. Furthermore, response biases would apply equally to the two conditions in Experiment 1. As the results were analysed in terms of the difference between these two conditions, little impact on the conclusion is expected.

## Discussion

In this study, it was demonstrated that the rated difficulty of extracting a simple melody from a background of random distracter notes increased as the overlap between melody and distracter notes increased. It was also shown that the presence of visual cues showing the entire melody, as well as the exact melody note playing at any instant, could reduce the rated difficulty of segregating the melody. It was found that musical training was not necessary to gain an advantage from the visual stimuli. This effect was relatively strong in listeners without musical training, however those with musical training received no additional benefit from the visual cues. These results indicate that visual cues could potentially help to restore part of the musical information degraded by hearing loss, and that no special training may be required to make use of these visual cues. Long-term musical training also reduced the rated difficulty, but provided no extra benefit in utilising the visual cues.

### The effect of pitch overlap

The task of separating a melody from a complex musical context is often required in many types of music appreciation. For example, in a piece of solo piano music, there is often a melody line carried by the right hand, and various accompaniments in the left hand. The ability to separate the melody from the accompaniment is vital to appreciating the intent of the composer and perceiving the affective impact of the music. In a pioneering study, Dowling [27], [33] introduced an interleaved-melody task as a means of investigating stream segregation. In these initial experiments, it was found that the ability to segregate pairs of known melodies depended on the pitch overlap between the melodies. It was possible to identify familiar melodies when there was a separation between them, but the task became more difficult when the melodies overlapped. In Dowling's [27] experiments, the pitch overlap between the two melodies was gradually decreased. In the first trials, the two melodies were completely overlapping, and participants were unable to name either melody. In the current experiment, a single four-note melody was presented against a background of random interleaved distracter notes; however, the results were very similar to Dowling's experiments with two interleaved melodies. When there was total overlap between melody and distracter notes, participants rated the melody extraction task as very difficult or impossible. As the overlap decreased, difficulty ratings also decreased. At the point where the distracter notes were just overlapping the melody (with the highest possible distracter note at the same height as the lowest melody note), participants were on average rating the difficulty at 39% of the maximum difficulty. With no overlap (and up to an octave separation), participants had no difficulty segregating the melody. It is misleading to compare absolute scores between this study and the Dowling studies, as in the latter, the task was a single identification of a melody instead of a continuous rating, and only a small part of the melody may have been needed for correct identification. In the current study listeners were asked to report upon the perception of all the notes. The use of random distracter notes rather than a second melody may also have made the task in the current experiment more or less difficult.

### The Effect of Visual Cues

**Figure 4:**
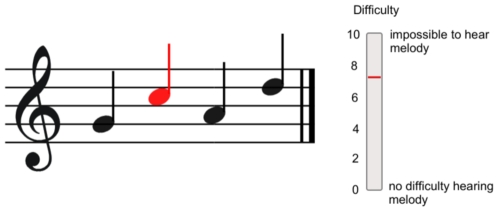
Visual cue of Experiment 1. A screenshot showing the visual cue (left) and the response indicator (right). Each note of the melody turned red as it played.

The key finding in the current study was the reduction of melody extraction difficulty when visual cues were provided. The visual cues took the form of a musical stave which was presented on a screen immediately in front of the participants (see Figure 4–method section). The stave showed the four melody notes and was animated such that each note in the melody turned red as it played. When the visual cue was present, non-musicians showed a 14% reduction in difficulty ratings averaged across all overlap levels of the experiment.

Visual cues have been shown to influence auditory streaming previously [16]. The current results extend this finding to the case of melody segregation, by showing that visual cues can reduce the difficulty of extracting a melody from background notes. Whether the visual effect on streaming is a result of improved encoding of acoustic features in the brainstem, or due to more top-down effects of the visual stimulus, is currently unknown, and a topic for further investigation.

As well as affecting auditory streaming, the effect of visual stimuli on auditory processing has been described at low levels in the brain. It has been shown that visual cues can improve the encoding of pitch and timbre in the auditory brainstem, particularly in musicians [34], [35]. The improvement in representations of these acoustic features in the brainstem may lead to more salient perceptual differences between sounds. As auditory stream segregation is based on pitch and timbre differences, this mechanism could possibly explain the effects of visual stimuli found in Rahne *et al* [16] as well as the current experiment. However, in the current experiment, it is still unclear why individuals with musical training did not benefit from the visual cues.

### The effect of music training

In the current experiment, musicians generally rated the task as less difficult than those without musical training when no visual cues were present. This result supports previous findings [17], [25], [26], [30]. However, previous work has also suggested that musicians use visual information more effectively than non-musicians to represent low-level features of sound [34], [35], and thus it was expected that musicians would gain more from the visual cues in the current experiment. However, in the current experiment, musicians found the task no less difficult than non-musicians when visual cues were provided. In order to maximise any potential effect, the visual stimulus was designed to take the form of a musical stave showing the melody notes–whereas the musicians had many years of experience with this type of cue, the non-musicians had very little experience (although all participants were instructed that notes low on the stave were low-pitched and high notes high-pitched). This finding cannot be explained by floor effects as when the melody and the distracter range totally overlapped, the musician group still found the task difficult, and there was still considerable room for reductions in difficulty.

One possibility is that musicians, although they are highly trained at reading musical scores, are also trained to read ahead or, in some cases, ignore the score. An animated score such as provided in the current experiment would be very unusual in most music practice or performance settings. While performing, musicians may take most of their visual cues from sources other than the score. In the cases where musicians do read the score, they are most likely reading ahead of the current position of the sound, and would almost certainly not read each note as it was played. In a task where concentration on separating difficult musical sources is required, highly trained musicians may first look for other more immediately salient visual cues, such as the movements of the conductor or other performers. If these cues are unavailable, they may ignore static visual cues such as the score, and solve the problem purely through audition. Further work is required to assess the best visual representations to assist with stream segregation in musical tasks.

### The effect of Context

The statistical analysis revealed a significant interaction between the context and the level. This complex interaction might be due to the combination of different phenomena. The following paragraphs will propose some possible explanations. When the note range of the distracter notes totally overlapped the melody, it was easier to segregate the melody if the distracter overlap had been gradually increased (INC blocks) toward that level, compared to the same overlap level in DEC blocks, where the melody and distracter note range was initially overlapping. This result can be partly explained by the well known build-up effect [1]. According to this phenomenon, when a new sequence is presented to a listener, the initial percept will most likely tend toward fusion. Then after several seconds, the sequence will either still be perceived as fused or will change toward segregation. In the current experiment, the build-up of streaming would have occurred at the beginning of each block. In DEC blocks, the build-up would thus occur during the most difficult part of the block–when the distracter notes completely overlapped the melody–and may have thus further increased difficulty ratings. However, this build-up effect is usually found to occur over a timescale of around 10 seconds [14], and in the current experiment, the duration of each overlap level was 16 seconds, therefore reducing the impact of any build-up effects.

There has been very little research investigating the effect of context in the segregation of melodies from background notes; however, the concept is similar to the gestalt principle of ‘emergence’. The typical example of emergence is in vision–a photograph of a spotty Dalmatian is degraded such that all the elements, including the dog, are made up of black or white spots [36]. When looking at the photo, one gradually sees the appearance of the dog, despite the fact that no outlines or other visual features of the dog are present. In a similar manner, a melody embedded in background notes can seem to emerge from the background notes. The effect of musical context on the difficulty of segregating a melody from background notes may be an important factor to consider for composers.

On the other hand, when the difference between the melody and the distracter notes was within a “bistable region,” when the listener's perception could switch easily between one or two streams, ratings were higher for INC blocks compared with DEC blocks. This result is consistent with the “contrastive context effect” found by Snyder *et al.* [28], [29]. At each level of the INC block, listeners were exposed to a previous level with a higher Δƒ between the melody and the distracter notes. According to Snyder *et al.*, this previous exposure increases the likelihood of fusion between each sequence and therefore increase the difficulty to perceive the melody.

### Implications for hearing impaired listeners

The current study was undertaken in order to assess whether visual cues may improve the ability to segregate musical sources for the hearing impaired, and whether training would be required to use these visual cues. When no visual cues were present. it was shown that those with musical training found the melody extraction task easier, indicating the effect that top-down processes like training can have on auditory stream segregation. It was also shown that visual cues could indeed reduce the difficulty of segregating a melody from background notes. These results demonstrate the possibility of using visual cues, either as part of an active listening device or as a training device, in order to improve music appreciation for the hearing impaired. More research is required to better understand the types of visual cues that will be most useful in this regard.

For those *with* musical training, there was no additional benefit of the visual cue, showing that there is no super-additive effect of both training and visual cues. The results also show that long-term training is not necessarily required in order to provide a useful enhancement of melody segregation by a visual cue. Providing a simple visual cue reduced the difficulty of extracting the melody to approximately the same degree as extensive musical training. These results show that there may be two approaches to improving stream segregation ability–the provision of assistive visual cues, and music training. However, training may still be of assistance in combination with visual cues, especially for those with hearing impairment. As hearing impaired listeners are often highly experienced with extracting auditory information from real-time visual cues (lip reading, subtitles, etc) it might be possible that they will gain even more from visual cues, and that this type of ‘real life’ training may be more effective than the formal music training tested here. Further studies investigating this effect in listeners with hearing impairment will be helpful in determining the extent of the benefits this approach to improving music appreciation for the hearing impaired may provide.

## Materials and Methods

### Ethics Statement

The experimental protocol conforms to The Code of Ethics of the World Medical Association (Declaration of Helsinki), and was approved by the Human Research Ethics Committee of the Royal Victorian Eye & Ear Hospital (Project 09-880H). Written informed consent was obtained from all participants involved in the study.

### Participants

Thirty-seven participants (20 females and 17 males) were recruited from the community using social networks and advertisements in music schools. Ages ranged from 18 to 45 years (mean  = 31.5, standard deviation  = 7.5). All participants reported normal hearing and normal or corrected-to-normal colour vision. Travel and lunch expenses were reimbursed $40 AUD. In order to assess participants' musical ability, four measures of musical activity were recorded: self-ratings on 0–5 scales for sight-reading ability and general musical aptitude, the number of hours of musical practice per week, and years of musical training.

### Stimuli

The melody and distracter notes were constructed using Matlab 7.5 and presented using MAX/MSP 5 through an M-AUDIO Firewire 48-kHz 24-bit sound card. Each note consisted of a 180 ms complex tone with 10 harmonics. Each successive harmonic was attenuated by 3 dB, and each note included a 30 ms raised-cosine onset and 10 ms offset. The notes were played from a loudspeaker (Genelec 8020APM) positioned on a stand at the listener's ear height, 1 m from the listener's head. Each note was equalised in loudness to 65 phons according to a loudness model [37].

The participants were exposed to a series of notes with each note onset presented every 200 ms. Within this series of notes was a repeated four-note target melody and interleaved distracter notes. The target melody pitches (see Figure 1) were G, C, A, and D above middle C (midinotes 67, 72, 69, and 74 respectively). As the experiment was also designed to be performed by listeners with hearing impairment, the melody was composed of intervals large enough to be perceived by people with poor pitch discrimination (as it is often the case in cochlear implant listeners) while being small enough for the sequence to be grouped into a single stream (instead of 2 interleaved streams composed of the 2 low notes and 2 high notes). For convenience, note pitches are referred to throughout using standard midinote values–middle C is designated ‘midinote 60’, with each integer corresponding to a semitone change in pitch. Each distracter note value was randomly chosen from a pool of 12 consecutive midinotes spanning an octave. Throughout the experiment, the note range of this octave pool was gradually varied providing a range of melody-distracter separation, or overlap levels (as described in the Procedure). It is worth noting that as the distracter notes were chosen randomly from every possible midinote within the octave range, the distracter notes were not necessarily in the same tonality (key) as the melody. However, it has been shown previously [30], that tonality has little effect on the difficulty of extracting a melody from interleaved background notes.

The visual cue was generated with the software MAX/MSP 5. It consisted of a musical staff with the 4-note target melody depicted in standard musical notation (see Figure 4). Each note in the visual cue turned red as the appropriate melody note played. In this way, the visual cue depicted the shape of the whole melody, as well as the current note playing. The synchronisation of the auditory-visual cue was measured by recording the output of a light-sensitive diode as well as the audio output in a 2-track audio file sampled at 44.1 KHz. The visual cue led the auditory stimulus by 36 ms. To ensure participants did not have to look down at the response slider during the experiment, a visual depiction of the response slider was shown on the screen immediately to the right of the staff. The current position value of the slider was updated in real time and shown in red.

### Procedure

Two counterbalanced sessions were run for each participant–one with the visual cue present (Vision) and one without (No-vision). The participants were asked to rate the difficulty of perceiving the four-note melody continuously throughout each block using a variable slider on a midi controller (EDIROL U33). The slider was labelled from 0 (no difficulty hearing melody) to 10 (impossible to hear melody). Participants were instructed to move the slider to the “10” position if the melody was impossible to perceive and to the “0” position if the melody could be easily perceived.

Each session was divided into four blocks - two blocks where the overlap of the melody and distracter notes gradually increased (INC) and two where it gradually decreased (DEC). The overlap was varied in 20 levels from no overlap (plus a separation of one octave between the highest distracter note and the lowest melody note) to total overlap, and expressed as the midinote value of the highest note in the distracter range. In INC blocks, distracter notes were initially picked from the range of midinotes 45–56. This starting range provided an octave separation between the highest possible distracter note and the lowest melody note, and was selected to ensure that the melody was easily perceived for every participant. The range of possible distracter notes was then slowly increased until they completely overlapped the melody (midinote range 65 to 76). In each level, the melody was repeated 10 times (lasting 16 seconds). In DEC blocks, the distracter note range initially completely overlapped the melody (midinote range 65 to 76) and was decreased in twenty steps until it reached the minimum level (midinotes 45–56). The INC and DEC blocks provided different musical context for the melody. The procedure is illustrated in Figure 1.

Before each test session, the melody was presented 20 times without distracter notes; an INC practice block followed. During testing, each INC/DEC block was repeated twice, with INC-DEC-DEC-INC or DEC-INC-INC-DEC order counterbalanced across participants. The duration of each block was about 5 minutes, and each session lasted about 30 minutes.

In order to reduce possible pitch memory effects between Vision and No-vision sessions, a pitch increment, randomly chosen between 0 and 4 semitones, was added to all notes of the same session.
